# Acceleration of tissue phase mapping with sensitivity encoding at 3T

**DOI:** 10.1186/1532-429X-13-59

**Published:** 2011-10-12

**Authors:** Anja Lutz, Axel Bornstedt, Robert Manzke, Patrick Etyngier, G Ulrich Nienhaus, Wolfgang Rottbauer, Volker Rasche

**Affiliations:** 1 Department of Internal Medicine II, University Hospital of Ulm, Ulm Germany; 2 Philips Research North America, Briarcliff Manor, USA; 3 Medisys Research Lab, Philips Healthcare, Suresnes, France; 4 Karlsruhe Institute of Technology, Karlsruhe, Germany

## Abstract

**Background:**

The objective of this study was to investigate the impact of sensitivity encoding on the quantitative assessment of cardiac motion in black blood cine tissue phase mapping (TPM) sequences. Up to now whole volume coverage of the heart is still limited by the long acquisition times. Therefore, a significant increase in imaging speed without deterioration of quantitative motion information is indispensable.

**Methods:**

20 volunteers were enrolled in this study. Each volunteer underwent myocardial short-axis TPM scans with different SENSE acceleration factors. The influence of SENSE acceleration on the measured motion curves was investigated.

**Results:**

It is demonstrated that all TPM sequences with SENSE acceleration have only minimum influence on the motion curves. Even with a SENSE factor of four, the decrease in the amplitude of the motion curve was less than 3%. No significant difference was observed for the global correlation coefficient and deviation between the motion curves obtained by the reproducibility and the SENSE accelerated measurements.

**Conclusions:**

It is feasible to accelerate myocardial TPM measurements with SENSE factors up to 4 without losing substantial information of the motion pattern.

## Background

Quantification of myocardial motion provides insight in the myocardial mechanics and enables a more detailed assessment of motion abnormalities in several cardiac diseases such as cardiac insufficiency. Among other imaging modalities like echocardiographic tissue Doppler imaging (TDI) [1], cardiovascular Magnetic Resonance (CMR) has proven its value for noninvasive assessment of global and regional cardiac function [2-5]. Myocardial motion can be quantified with CMR using a variety of techniques including tagging [6,7], strain encoding (SENC) [8], displacement encoding with stimulated echoes (DENSE) [9], and phase contrast velocity encoding (tissue phase mapping, TPM) [2,10-14].

TPM directly encodes the myocardial velocity by the application of bipolar gradients and enables the quantitative assessment of three-directional motion. The spatial resolution of TPM is only limited by the voxel size. Despite the obvious advantages of TPM, its wide application is still limited by the long acquisition times, which precludes large volume coverage at sufficient spatial resolution and may cause image degradation due to irregular respiratory and cardiac motion [15]. For wide clinical applications an acceleration of acquisition time without information loss is mandatory.

Several generic methods accelerating the image acquisition have been introduced. Local imaging techniques reduce the field-of-view (FOV) to a confined area in order to reduce the scan time [15-17]. Clinical applications of these techniques are still limited due to their sensitivity to patient motion and the required complicated planning of the anatomy. Accelerating techniques like view sharing [18,19] and k-t BLAST [20] exploit temporal correlations. View sharing has shown to measure an accurate velocity profile with a gain of acquisition speed in the order of 37.5% [19]. Accelerating techniques like k-t BLAST allow higher accelerating factors. A disadvantage of the k-t BLAST algorithm is a reduction of peak velocity values due to temporal smoothing, which was observed in quantitative phase contrast angiography (PCA) [21-23] as well in myocardial tissue phase mapping [24].

Parallel imaging techniques exploiting coil sensitivities for unaliasing like sensitivity encoding (SENSE) [25] and generalized auto-calibrating partially parallel acquisitions (GRAPPA) [26] enable high acceleration factors. No temporal smoothing is expected. The aim of this work was to quantify the feasibility of using SENSE for tissue phase mapping of the left ventricle. An analysis of the influence of SENSE on TPM based flow quantification was performed and investigated for different accelerating factors. A case example is added to show the possibility of the combination of SENSE with TPM to measure the left-ventricular myocardial motion in 3D.

## Methods

### Volunteers

A total of 20 volunteers (8 females, 12 males, age 30 ± 10 years) were enrolled in this study. One additional volunteer was examnied to show the possibility of the use of SENSE to enable 3D TPM imaging (male,24 years). The study protocol was approved by the local ethics committee. All volunteers provided written informed consent prior to the CMR examination.

### Data acquisition

Image acquisition was performed on a 3T whole body MR scanner (Achieva 3.0T, Philips, Best, The Netherlands) with a 32 [2 × 4 × 4] channel phased array cardiac coil.

A coil-sensitivity reference scan was acquired for obtaining the coil sensitivity profiles as prerequisite for the subsequent SENSE reconstructions. Breath-hold cine cardiac two- and four chamber views were acquired to define the equatorial short-axis image orientation.

The TPM acquisition was performed for one equatorial short axis applying a black blood prepared [14,27], respiratory navigated, segmented and velocity encoded cardiac triggered gradient echo sequence. The acquisition parameters are listed in table 1.

**Table 1:** Acquisition protocol for the 2D and 3D data acquisitions

Acquisition parameter	Value	
sequence type	velocity encoded segmented gradient echo	velocity encoded segmented gradient echo
TR/TE	7 ms/4.7 ms	7.1 ms/4.9 ms
FOV	340 × 340 mm^2^	380 × 380 mm^2^
flip angle	15°	15°
k-lines per segment	3 + 1 startup echo	3
acquisition matrix	172 × 168	128 × 124
spatial resolution	2 × 2 × 8 mm^3^	3 × 3 × 3 mm^3^
		(with over-contiguous slices)
black blood mode	alternating	alternating
number of slices	1	21
cardiac phases at 60 bpm	21	23
phase interval	40 ms	37.3 ms
VENC	30 cm/s	20 cm/s
navigator duration + evaluation	15.5 ms + 5 ms	15.5 ms + 5 ms
number of chunks		3 (to enable black blood suppression)
RR interval	90%	90%
SENSE acceleration factor R	1,2,3,4	4
nominal scan duration [min]	3:45, 1:57, 1:17, 0:57 (corresponding to R)	15:30

Isotropic velocity encoding of 30 cm/*s *was performed in a Hadamard fashion by a four-point velocity vector method [28,29]. To improve the temporal resolution, the different flow encoding directions were encoded in subsequent heart beats [14,24].

Black blood preparation was performed by two presaturation slabs of 40 mm thickness with 8 mm distance on either side of the imaged slice in order to reduce flow artifacts [14,27,30]. The duration of the saturation module consisting of the saturation pulses and spoiler gradients was 12 ms. The saturation slabs were applied alternating in subsequent cardiac phases to avoid SAR limitations at 3T [31]. Thus the effective distance between two saturation pulses at the same position was 80 ms. Further SAR optimization was achieved by limiting the B1-amplitude to 8 *μ*T in all experiments. Further reducing the B1-amplitude prolonged the presaturation pulse part of the sequence, whereas higher B1-amplitudes resulted in too high SAR demands.

For respiratory motion compensation, conventional navigator-gating and tracking with an acceptance window of 10 mm was performed applying a pencil beam navigator through the dome of the right hemi-diaphragm [32]. The navigator was applied at each start of the cardiac cycle [33]. The navigator duration was 15.5 ms, the navigator evaluation time was 5 ms. Prospective gating was performed since it reveals superior image quality than retrospective gating [34]. Cardiac triggering was performed using a vector electrocardiogram.

In each volunteer 5 different sequences were performed: two scans without SENSE acceleration for the assessment of the reproducibility and 3 scans with SENSE acceleration factors R of 2,3 and 4. The direction of SENSE was chosen dependent on the orientation of the heart either in RL (8 volunteers) or SI direction (12 volunteers).

For a pulse rate of 60 beats per minute 21 heart phases could be acquired. The nominal imaging times were 3 : 45 minutes without SENSE acceleration, 1 : 57 minutes with a SENSE factor of 2, 1 : 17 minutes with a SENSE factor of 3 and 57 seconds for a SENSE factor of 4, so that the nominal acceleration factors are given by 1.92, 2.92 and 3*:*95 for a SENSE factor of *R *= 2, 3 or 4 respectively.

One volunteer underwent a black blood prepared, respiratory gated, cardiac phase resolved 3D anatomical and velocity encoded acquisition. For maximal acceleration a SENSE acceleration factor of *R *= 4 was chosen. Data were acquired on a 3T whole body MR scanner (Achieva 3.0T, Philips, Best, The Netherlands) with a 32 [2 × 4 ×4] channel phased array cardiac coil. The acquisition protocol is listed in table 1.

### Data analysis

The TPM MR images were analyzed by an in-house developed MATLAB software (R2008a; Mathworks, Natick, Mass). For each scan the segmentation of the myocardium was performed automatically relying on active-contour techniques by incorporating a shape model. After the segmentation of the first phase, the information was propagated through the entire sequence by tracking profile intensities [35,36].

Before quantification of the resulting myocardial velocities, a background phase error correction was performed using a linear fit to the phase of static tissue [37].

The radial (towards the center of the blood pool) and longitudinal (towards the apex of the heart) velocity curves were calculated. Velocity-time curves for either direction of motion were generated using the average myocardial velocity of the respective slice. Prior to data analysis the velocity data acquired over time were interpolated by cubic splines to provide a continuous velocity profile. Physiologically, the accumulated phase over the entire heart cycle must result to zero. To reduce remaining phase errors after the linear background phase error correction described above, in a subsequent correction step the resulting velocity curves were shifted accordingly to meet the physiological conditions.

The systolic and diastolic peak velocities v_p,sys _and v_p,dias _were determined and the velocity range Δv = v_p,sys _- v_p,dias _was calculated for each sequence. Bland-Altman analysis was performed for the velocity differences Δv and the differences between Δv with and without SENSE acceleration was denoted as Δv Diff. The peak factor $\text{PF}\left(\text{seq}.1,\text{ seq}.2\right)=\frac{\Delta {\text{v}}_{\text{seq}.2}}{\Delta {\text{v}}_{\text{seq}.1}}$ was calculated. The quality of the resulting velocity curves was additionally quantified by the normalized root mean square deviation (nRMSD(seq. 1, seq. 2)) between the velocity curves obtained by the SENSE accelerated and non-accelerated technique and the correlation coefficient s c(seq:1, seq.2). Thereby, the correlation coefficient is given by

(1) $$c=\sum_{i=1}^{N}\left[\left(\frac{v_{\text{i,seq}. 1}-{\bar{v}}_{\text{seq}.1}}{\sigma_{\text{seq}.1}}\right)\left(\frac{v_{\text{i,seq}. 2}-{\bar{v}}_{\text{seq}.2}}{\sigma_{\text{seq}.2}}\right)\right],$$

where *v*_i,seq.1 _and *v*_i,seq.2 _are the spline interpolated data at different time steps (step size: 0.01 ms).

For the regional analysis of velocity information, the velocity range, peak factor an systolic and diastolic peak velocities are determined for six segments of the investigated equatorial slice from anteroseptal to anterior.

The times to the minima radial and longitudinal velocity, t_r,dias _and t_l,dias _were determined for each acquisition technique. Bland-Altman analysis was performed for t_r,dias _and t_l,dias _and the mean time differences Δt_r,dias_(seq.1, seq.2) = t_r,dias,seq.2 _- t_r,dias,seq.1 _and Δt_l,dias_(seq.1, seq.2) = t_l,dias,seq.2 _- t_l,dias,seq.1 _and their standard deviations over all volunteers were compared.

For assessment of the inherently reduced signal-to-noise ratios (SNR) in parallel imaging, SNR maps of the anatomical and velocity encoded data were calculated. Local SNR values were derived from the mean value of a 3 × 3 matrix divided by its standard deviation. SNR values were calculated for the whole myocardium and the respective regional 6 segments.

For the evaluation of significances a paired two-tailed student's t-test was performed. P-values below 0.05 were considered to be significant. The 3D anatomical + 3D velocity encoded + time acquisition was analyzed regarding the radial and longitudinal motion at different position of the left ventricle.

## Results

The scan protocol could be finished in all volunteers. The navigator gating efficiencies of this study were 68.8% ± 14.2% for no SENSE (1), 72.6% ± 12.0% for no SENSE (2), 71.5% ± 12.9% for SENSE with *R *= 2, 70.0% ± 15.3% for SENSE with *R *= 3 and 71.9% ± 13.6% for SENSE with *R *= 4 and did not show any significant differences for all acceleration factors.

**Figure 1:**
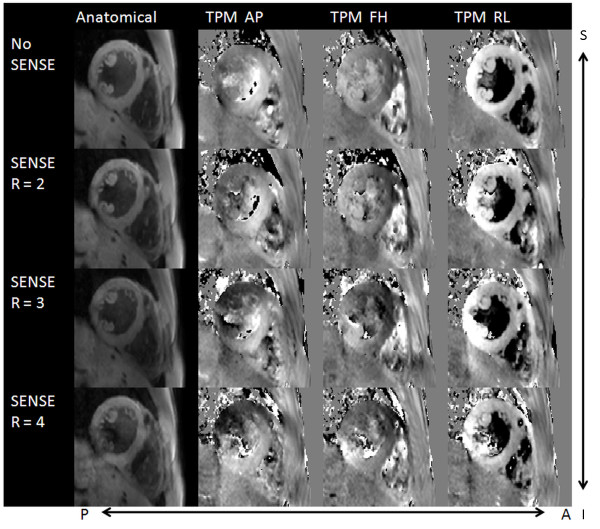
**Anatomical and phase contrast images of the left ventricle with different SENSE acceleration factors**. Anatomical and phase contrast images of a short-axis TPM measurement of the left ventricle without SENSE acceleration and with SENSE acceleration factors 2 - 4. The velocity images are divided into 3 columns for each direction of motion (right-left, anterior-posterior and superior-inferior). Visually only small intensity differences can be revealed in the anatomical images, whereas a decrease of the velocity magnitude was only observed for *R *= 4 in the velocity encoded images.

Figure 1 shows anatomical and velocity encoded images of a myocardial short-axis scan acquired without SENSE and with SENSE acceleration factors *R *= 2 - 4 exemplary for one volunteer. Visually, only small intensity differences can be appreciated in the anatomical images, whereas a decrease of the velocity magnitude could only be observed for *R *= 4 in the velocity encoded images.

**Figure 2:**
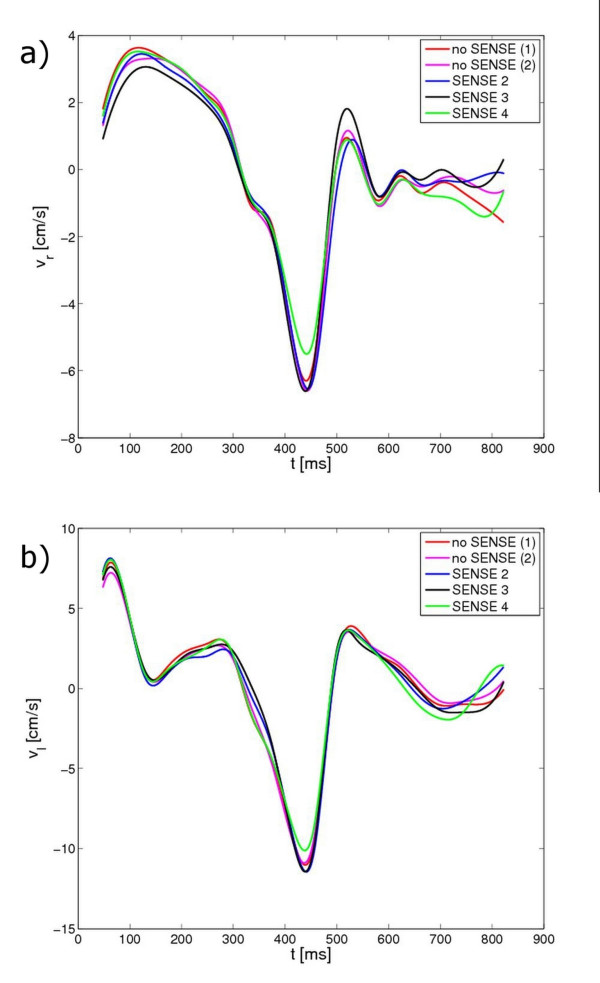
**Radial and longitudinal velocity curves over time exemplary for one volunteer**. Radial (a) and longitudinal (b) velocity curves over time for the different sequences no SENSE (1), no SENSE (2), SENSE 2, SENSE 3 and SENSE 4 exemplary for one volunteer.

Figures 2a) and 2b) show the respective radial and longitudinal velocity curves over time for the investigated sequences. Visually, only for an acceleration factor of four a small decrease in the peak amplitude was observed. Table 2 provides the radial and longitudinal peak velocities separately for systole and diastole and the velocity ranges. Significant differences between the accelerated and non-accelerated values were only obtained for the longitudinal diastolic peak velocity for an acceleration factor of *R *= 4. Table 3 provides the peak factors PF_r _and PF_l _for the different SENSE acceleration factors for the radial and longitudinal velocity of the myocardium. A small but significant (p-value *<*0.05) reduction of the PF was obtained in the case of the radial motion for a SENSE accelerating factor R of 2 and 4. The lowest peak-factor value obtained with SENSE was 3% lower than the optimal value of 1.

**Table 2:** Radial and longitudinal systolic and diastolic peak velocities and velocity ranges for the sequences no SENSE (1), no SENSE (2) and SENSE with R = 2 - 4

**seq**.	v_r,sys_	v_r,dias_	Δv_r_	v_l,sys_	v_l,dias_	Δv_l_
no SENSE (1)	2.90 ± 0.55	-5.03 ± 0.53	7.93 ± 0.83	6.08 ± 2.24	-8.58 ± 2.58	14.66 ± 4.28
no SENSE (2)	3.02 ± 0.63	-5, 15 ± 0.72	8.17 ± 1.00	6.18 ± 2.36	-8.46 ± 2.69	14.63 ± 4.44
SENSE 2	2.83 ± 0.56	-4.99 ± 0.68	7.82 ± 0.98	6.14 ± 2.26	-8.76 ± 2.72	14.91 ± 4.46
SENSE 3	2.94 ± 0.65	-4.89 ± 0.66	7.83 ± 1.11	6.04 ± 2.21	-8.38 ± 2.61	14.41 ± 4.22
SENSE 4	2.93 ± 0.57	-4.78 ± 0.58	7.71 ± 0.96	6.07 ± 2.21	-8.15 ± 2.29	14.22 ± 4.06

**Table 3:** PF values of the sequence no SENSE (1) (seq. 1) and the sequences no SENSE (2) and SENSE with R = 2 - 4. (seq. 2)

seq. 1	seq. 2	PF_r _	ΔPF_r _	PF_l _	ΔPF_l _
no SENSE (1)	no SENSE (2)	1.03	0.09	1.00	0.10
no SENSE (1)	SENSE 2	0.99	0.08	1.02	0.10
no SENSE (1)	SENSE 3	0.99	0.07	0.99	0.12
no SENSE (1)	SENSE 4	0.97	0.07	0.98	0.12

The results of the Bland-Altman analysis of the velocity range Δv are shown in table 4. There were no significant differences between Δv_r _Diff and Δv_l _Diff obtained by the reproducibility measurement and the SENSE accelerated measurements.

**Table 4:** Results of the Bland-Altman analysis of the velocity ranges *v_r_*and *v_l_*

R(seq. 1)	R(seq. 2)	Δv_r _Diff	Δv_l _Diff
1	1	-0.20 ± 0.71	0.02 ± 1.27
1	2	0.16 ± 0.62	-0.25 ± 1.19
1	3	0.14 ± 0.59	0.25 ± 1.54
1	4	0.27 ± 0.56	0.43 ± 1.62

**Figure 3:**
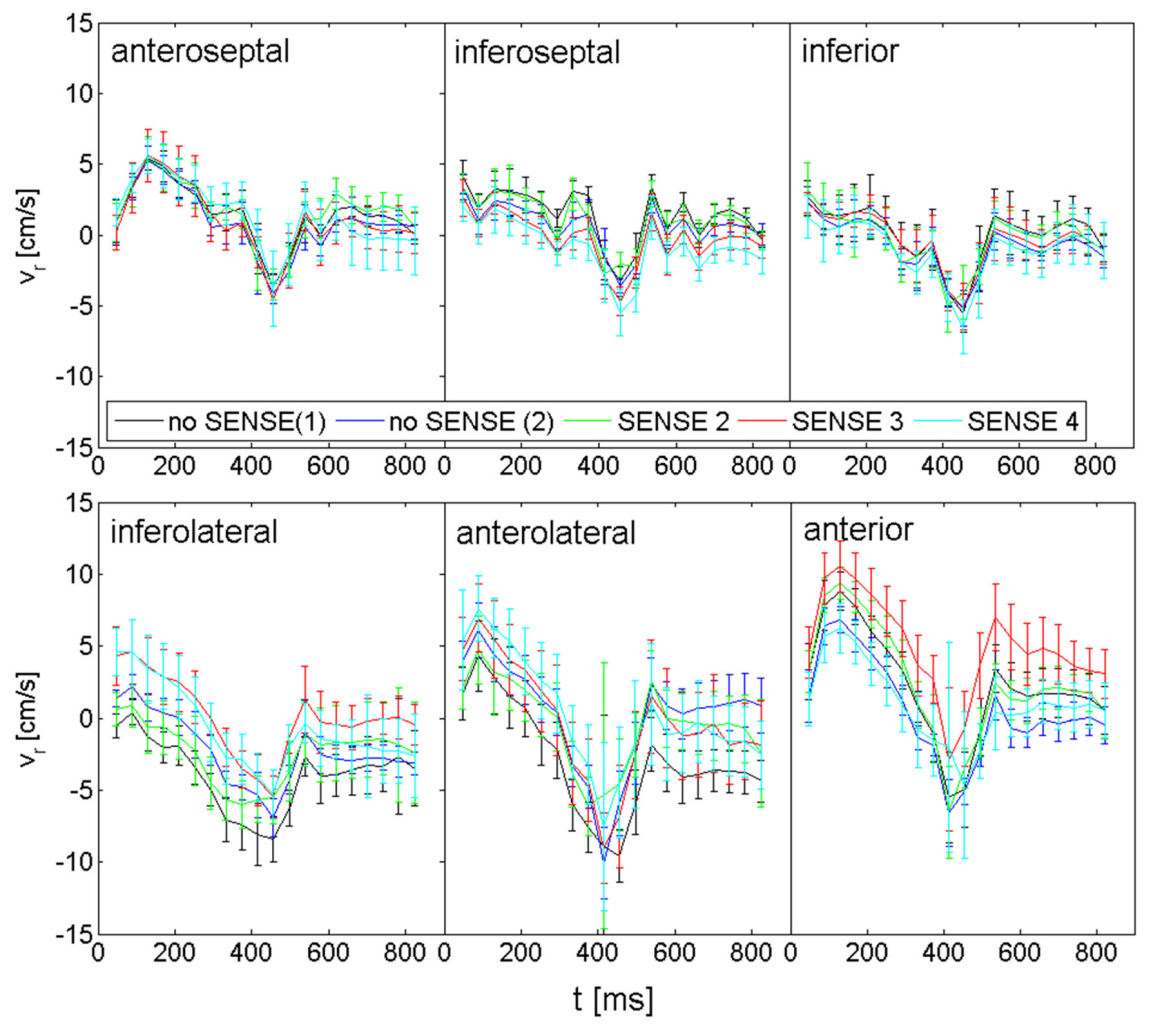
**Segmental radial velocity curves over time exemplary for one volunteer**. Regional radial velocity curves over time for the different sequences no SENSE (1), no SENSE (2), SENSE 2, SENSE 3 and SENSE 4 exemplary for one volunteer.

**Figure 4:**
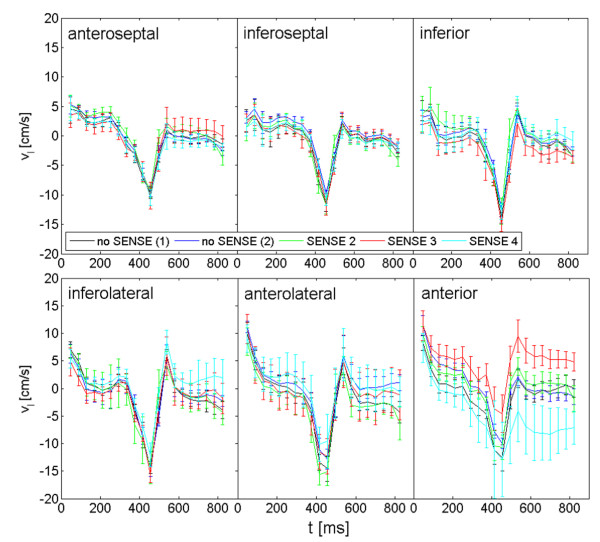
**Segmental longitudinal velocity curves over time exemplary for one volunteer**. Longitudinal radial velocity curves over time for the different sequences no SENSE (1), no SENSE (2), SENSE 2, SENSE 3 and SENSE 4 exemplary for one volunteer.

Figures 3 and 4 display the radial and longitudinal velocities over time for each segment exemplary for one volunteer. The segmental analysis reveals some deviations between the different acquisitions. However, all deviations appear to be within the reproducibility of the investigated technique.

**Figure 5:**
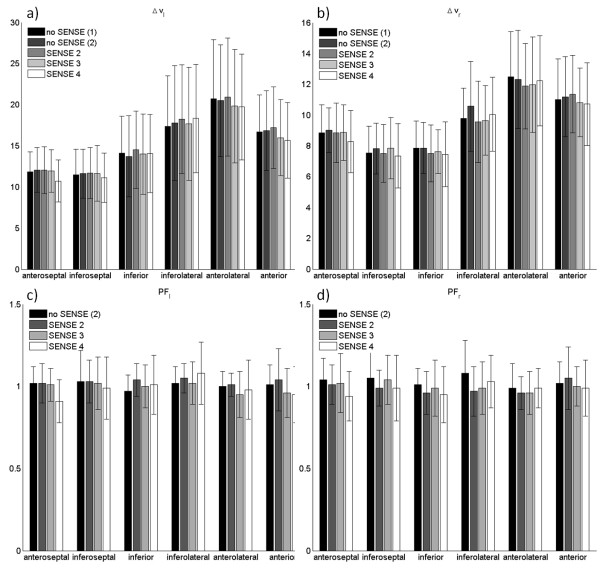
**Longitudinal and radial velocity ranges and peak factors averaged over all volunteers**. The longitudinal (a,c) and radial (b,d) velocity ranges (on the top) and peak factors (at the bottom) are displaced for each segment.

Figures 5 displays the radial and longitudinal velocity ranges and peak factors. Significant deviations were found for the longitudinal (R = 4, anteroseptal) and radial (R = 2, anterolateral) velocity range and peak factor (R = 4, PF*_r _*and PF*_l_*, anterolateral), and radial (R = 2,4, anterolateral) and longitudinal (R = 4, anteroseptal, anterolateral and anterior) diastolic peak velocities. Thus, for most acceleration factors and segments, the longitudinal and radial velocity ranges and peak factors are not altered.

**Figure 6:**
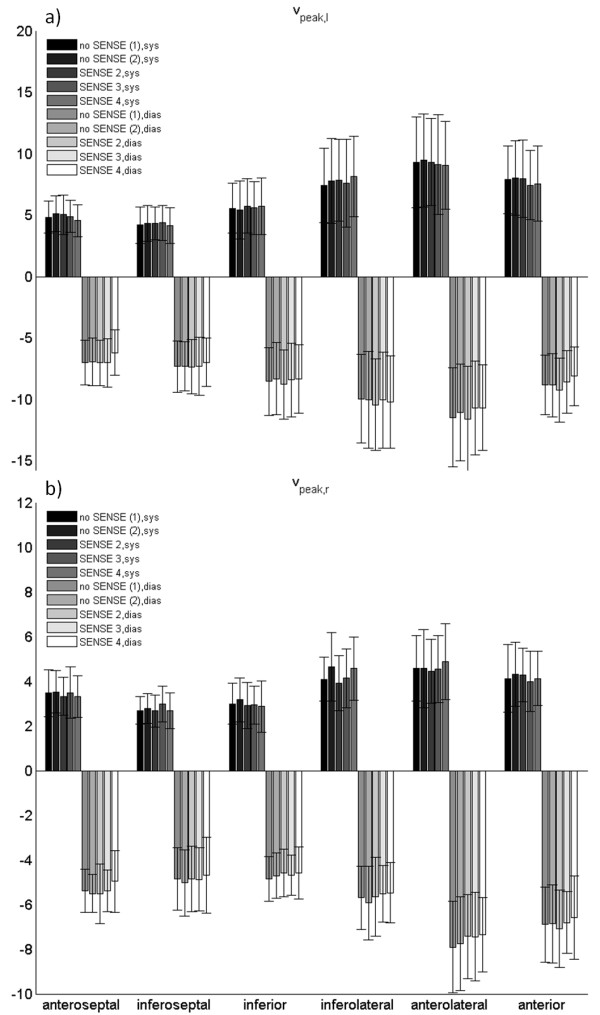
**Longitudinal and radial peak velocities averaged over all volunteers**. The longitudinal (a) and radial (b) peak systolic and diastolic velocities are displaced for each segment.

Figure 6 displays the radial and longitudinal systolic and diastolic peak velocities for the different segments. The absolute value of peak velocities was only significantly reduced for *v*_r,dias _in the anterolateral segment for acceleration factors *R *= 2, 4 and for *v*_l,dias _in the anteroseptal, anterolateral and anterior segment for an acceleration factor of *R *= 4. The mean nRMSD_r _and nRMSD_l _values and their standard deviations are listed in table 5. A significant difference between the obtained mean nRMSD values was not observed.

**Table 5:** nRMSD*_r _*and nRMSD*_l _*values between the sequence no SENSE (1) (seq. 1) and the sequences no SENSE (2) and SENSE with accelerating factors from 2 - 4 (seq. 2)

R(seq. 1)	R(seq. 2)	nRMSD_r_[%]	nRMSD_l_[%]
1	1	5.1 ± 2.4	4.6 ± 1.9
1	2	4.9 ± 1.4	4.4 ± 1.7
1	3	5.4 ± 2.4	4.4 ± 2.4
1	4	5.2 ± 2.3	4.7 ± 1.6

Table 6 provides the correlation coefficients c_r _and c_l _between the reproducibility and accelerated scans. No significant difference between these values could be obtained for the different acceleration techniques in the global analysis over all segments. The segmental analysis only reveals a significant difference of the longitudinal correlation coefficient for the anterior segment with an acceleration factor of 4.

**Table 6:** Correlation coefficients c_r _and c_l _between no SENSE (1) (seq. 1) and no SENSE (2), SENSE 2, SENSE 3 or SENSE 4 (seq. 2)

R(seq. 1)	R(seq. 2)	c_r_	c_l_
1	1	0.98 ± 0.02	0.98 ± 0.02
1	2	0.98 ± 0.01	0.98 ± 0.01
1	3	0.98 ± 0.02	0.98 ± 0.02
1	4	0.98 ± 0.03	0.98 ± 0.01

The time to the diastolic peak radial and longitudinal velocities was calculated for all volunteers. The results of the Bland-Altman analysis for the mean time differences Δ*t*_r,dias _and Δ*t*_l,dias _over all volunteers and their standard deviations are provided in table 7. Significant differences between the relative time differences obtained by the reproducibility measurement and the time differences between the accelerated and non-accelerated SENSE TPM measurement were not observed.

**Table 7:** Results of the Bland-Altman analysis for Δt_r,dia__s _and Δt_l,dias_

R(seq. 1)	R(seq. 2)	Δt_r,dias[ms] _	Δt_l,dias[ms] _
1	1	1.00 ± 9.26	1.72 ± 16.60
1	2	0.48 ± 9.24	1.73 ± 13.12
1	3	2.50 ± 7.70	-0.89 ± 5.45
1	4	2.41 ± 7.51	-1.82 ± 13.60

**Figure 7:**
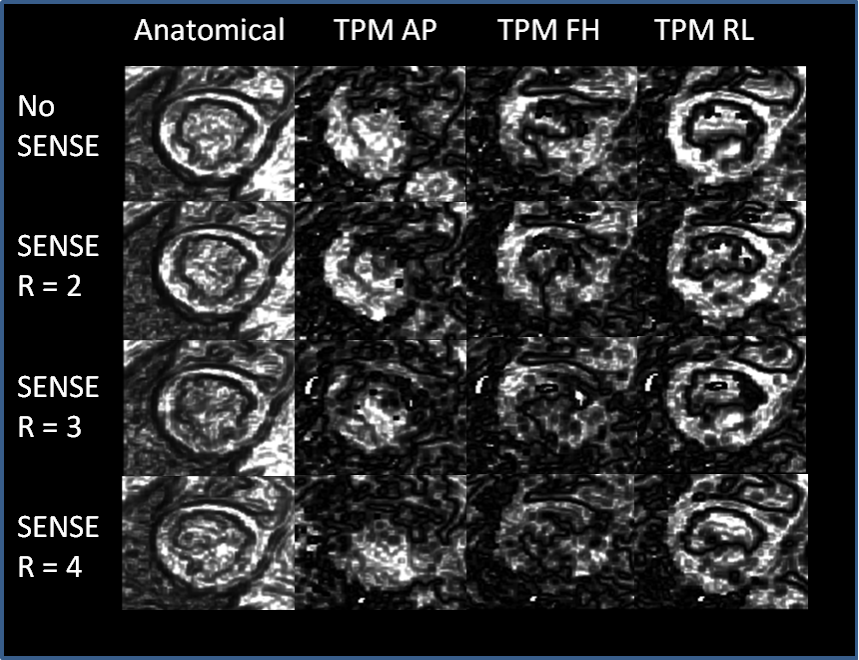
**Anatomical and phase contrast SNR maps of the left ventricle with different SENSE acceleration factors**. Anatomical and phase contrast SNR maps of the left ventricle without SENSE acceleration and with SENSE acceleration factors 2 - 4. The velocity images are divided into 3 columns for each direction of motion (anterior-posterior, superior-inferior and right-left). Visually the SNR decreases with increasing acceleration factor.

**Figure 8:**
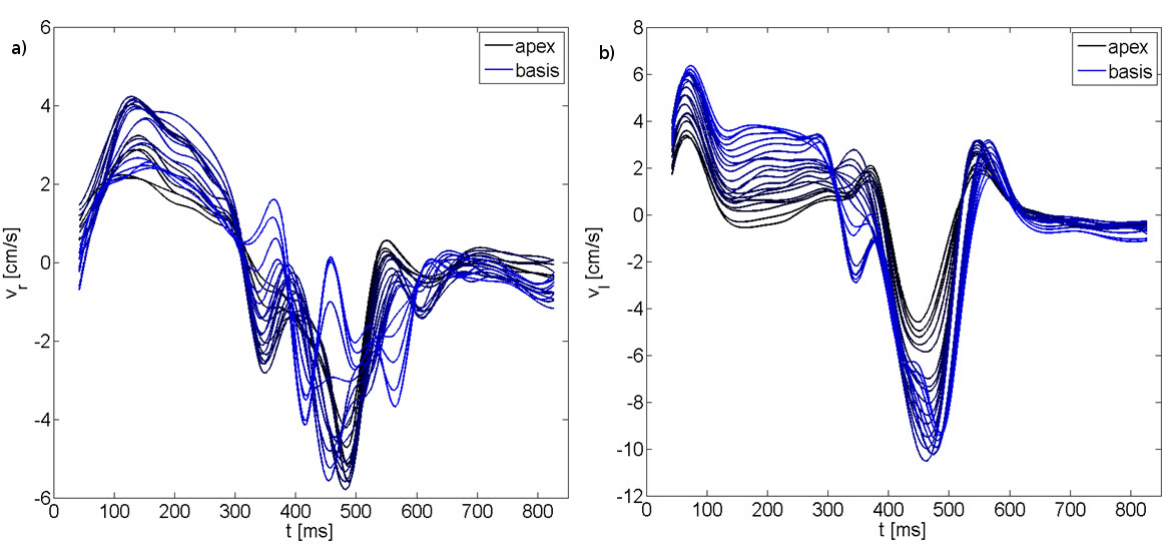
**Radial and longitudinal velocity curves in 3D exemplary for one volunteer**. Radial (a) and longitudinal (b) velocities over time are displaced from apex to basis with the 3D anatomical + 3D velocity + time data acquisition with a SENSE acceleration factor of 4. A strong variation appears dependent on the position of the left ventricle.

Figure 7 displays the SNR maps for the anatomical and velocity encoded images. A steady decrease in the SNR with increasing acceleration can be appreciated. Table 8 provides the mean SNR values of the entire myocardium and for all equatorial AHA heart segments. The decrease of SNR was statistically significant. Application of the investigated technique to the three-dimensional coverage of the left ventricle is provided in Figure 8. The continous change of the radial and longitudinal velocity patterns from the apex to the base can be clearly appreciated.

**Table 8:** Results of the SNR analysis for the anatomical and velocity encoded images

selected segments	no SENSE (1)	no SENSE (2)	SENSE 2	SENSE 3	SENSE 4
anatomical information					

all	32.36 ± 4.81	32.21 ± 5.00	27.64 ± 3.48	26.81 ± 4.43	23.91 ± 2.90
anteroseptal	39.23 ± 7.43	38.61 ± 6.77	33.02 ± 4.55	31.98 ± 6.00	28.73 ± 4.68
inferoseptal	37.22 ± 8.72	36.25 ± 8.70	31.21 ± 5.69	31.28 ± 7.08	27.65 ± 5.98
inferior	26.93 ± 5.52	27.06 ± 5.25	24.54 ± 4.19	23.81 ± 5.51	21.25 ± 4.66
inferolateral	31.28 ± 5.74	32.03 ± 7.25	27.73 ± 4.55	25.09 ± 5.81	22.92 ± 4.31
anterolateral	28.68 ± 5.13	28.58 ± 6.60	23.36 ± 4.65	22.67 ± 4.23	20.38 ± 4.81
anterior	31.14 ± 5.58	31.01 ± 5.63	26.09 ± 5.11	25.84 ± 5.47	22.41 ± 4.53

velocity information in AP direction					

all	6.73 ± 1.49	6.57 ± 1.28	5.23 ± 1.06	4.76 ± 1.09	4.30 ± 0.75
anteroseptal	5.94 ± 1.54	6.01 ± 1.42	4.96 ± 1.64	4.69 ± 1.38	3.95 ± 1.04
inferoseptal	5.93 ± 1.40	5.66 ± 1.32	4.67 ± 1.22	4.15 ± 0.97	3.92 ± 0.85
inferior	7.21 ± 1.86	6.80 ± 1.56	5.54 ± 1.32	4.79 ± 1.24	4.45 ± 1.26
inferolateral	7.44 ± 2.60	7.24 ± 2.08	5.47 ± 1.89	4.63 ± 1.07	4.32 ± 1.16
anterolateral	6.99 ± 1.80	7.15 ± 1.96	5.50 ± 1.46	5.27 ± 1.67	4.75 ± 1.19
anterior	6.84 ± 2.23	6.55 ± 1.65	5.21 ± 1.46	5.03 ± 1.79	4.42 ± 0.87

velocity information in FH direction					

all	7.03 ± 1.61	7.01 ± 1.61	5.80 ± 1.35	5.12 ± 1.36	4.41 ± 0.92
anteroseptal	7.23 ± 1.44	7.09 ± 1.79	5.77 ± 1.62	5.23 ± 1.39	4.56 ± 1.11
inferoseptal	6.41 ± 1.79	6.24 ± 2.01	5.49 ± 1.58	4.68 ± 1.40	4.23 ± 1.18
inferior	7.21 ± 2.06	7.35 ± 1.77	5.90 ± 1.66	5.29 ± 1.32	4.57 ± 1.09
inferolateral	7.40 ± 2.61	7.68 ± 2.54	6.29 ± 1.87	5.47 ± 2.13	4.81 ± 1.49
anterolateral	6.94 ± 3.09	6.66 ± 2.84	5.42 ± 1.76	4.77 ± 2.06	4.09 ± 1.31
anterior	6.94 ± 1.72	6.98 ± 1.87	5.85 ± 1.95	5.18 ± 1.07	4.17 ± 0.99

velocity information in RL direction					

all	7.67 ± 1.84	7.54 ± 1.51	5.89 ± 1.42	5.51 ± 1.18	4.94 ± 1.10
anteroseptal	7.47 ± 1.96	7.56 ± 1.77	5.97 ± 1.24	5.91 ± 1.25	4.95 ± 1.28
inferoseptal	6.86 ± 1.90	6.64 ± 2.13	5.64 ± 1.74	5.00 ± 1.37	4.66 ± 1.13
inferior	7.95 ± 2.17	7.59 ± 1.68	6.31 ± 2.18	5.64 ± 1.72	5.29 ± 1.40
inferolateral	8.64 ± 2.29	8.86 ± 1.87	6.60 ± 1.84	5.93 ± 1.64	5.55 ± 1.48
anterolateral	7.58 ± 2.62	7.38 ± 2.38	5.43 ± 1.73	5.11 ± 1.38	4.66 ± 1.58
anterior	7.44 ± 2.44	7.18 ± 1.98	5.32 ± 1.65	5.39 ± 2.03	4.47 ± 1.50

Radial motion: In systole, all slice first move toward the center of the heart. The highest radial velocities occur in equatorial regions. In systole, apical slices perform a single outward motion, whereas for basal segments two peaks of outward motion can be observed.

Longitudinal motion: At the beginning of systole, apical, equatorial and basal slices move towards the apex of the heart. This motion is stronger for basal than for apical slices. At the end of systole, the velocity of the basal slices approach a plateau, whereas most apical slices reach small negative values. During diastole, all slices move back towards the basis. Again, this motion is more pronounced in the basis than in the apex. Finally, a short motion in opposite direction occurs in all regions.

## Discussion

The application of SENSE to accelerate the acquisition of TPM data for the quantification of myocardial velocities appears feasible. All investigated SENSE accelerating factors can be applied without fundamental loss of motion information of the myocardium. No significant differences were obtained for the nRMSD, the global correlation coefficient *c *and the temporal differences Δ*t_dias _*between the reproducibility and accelerated measurements and the absolute velocity differences Δ*v *for both the radial and longitudinal velocity curves. Significant differences were obtained for the peak-factor. This differences of the peak factor can be caused by the increased noise in the SENSE accelerated data, which is an inherently property of SENSE. Nevertheless, these differences were not clinical relevant (less than 3%). Delfino et al. found a mean decrease in diastolic peak velocity values in heart failure patients between 68% (longitudinal) and 13% (circumferential) in comparison to healthy volunteers [14]. Therefore, the use of SENSE for TPM imaging appears promising. In most segments, the analysis of the velocity ranges, peak factors and peak velocities obtains no significant deviations.

The major limitation of SENSE in tissue phase mapping is the inherent decrease of SNR. The presented data indicate that the reduction in SNR does not cause clinical relevant deviations of the velocity information. Deviations of the velocity range and peak velocities appear to be within the reproducibility of the underlying technique and hence no significant limitations are expected from using SENSE. Whether the intrinsically lower SNR values at lower field strength will pose limitations remain to be investigated. Since in this study SENSE acceleration was restricted to the phase-encoding direction, the results should be representative also for coil arrays with a lower number of elements (e.g. a 6- or 12 channel coil) as long the acceleration factors does not exceed the number of receive coils in phase encoding direction and the resulting SNR is not limited by the performance of the coil.

This high performance of SENSE to measure accurate velocity profiles was also found in previous studies Investigating PC-CMR combined with SENSE in vessels [38,39]. GRAPPA, which uses correlations ink-space instead of image-space, has also shown high performance in PC-CMR acquisitions in vessels [40]. It can be assumed, that the accuracy of the measurement of myocardial velocity profiles with GRAPPA is similar to the accuracy of SENSE.

One method to even further accelerate TPM data acquisition would be the combination of SENSE with view sharing, thus exploiting correlation in both, k-space and time. Since the gain of acquisition speed in view sharing is based on less data acquisition in the outer part of k-space, no further velocity degradation would be expected.

In this study a temporal resolution of 40 ms was used. Higher temporal resolutions might increase the accuracy in the peak velocity quantification and can for example be obtained with view sharing [19], which also decreases scan time. Since the aim of this study was to investigate SENSE independent on other accelerating techniques, in this study view sharing was not applied.

A combination of k-t BLAST and SENSE for velocity encoded imaging was performed for blood flow measurements in the ascending aorta [22]. With an high acceleration factor of 8 a slow temporal low-pass filtering was obtained, whereas the flow measurement with an acceleration factor of 5 agreed to the reference flow measurement of this study. Further, it has been demonstrated, that there is only minor deviation of the myocardial motion for the k-t GRAPPA approach for an accelerating factor of 6 [41]. Therefore k-t SENSE might be used to further accelerate the TPM data acquisition.

A further method to accelerate TPM data acquisition with even higher acceleration factors would be the combination of SENSE with a temporal constraint k-t BLAST approach using principal component analysis (k-t PCA) [42]. First perfusion experiments in vivo show a higher performance of k-t PCA compared with k-t SENSE for high acceleration factors [42]. Future velocity encoded k-t PCA experiments of the human myocardium are necessary to validate the applicability of this technique.

In this study, the SENSE approach was only applied for accelerating the data acquisition. Alternatively, the gained acquisition time might be used to increase the spatial resolution or to encode more imaging slices. The combination of TPM and SENSE may provide wide application of myocardial motion measurements with sufficient spatial and temporal resolution in research as well as in clinical routine. Additionally it has The potential to enable 3D anatomical + 3D velocity encoded whole heart cine imaging, which is shown exemplary in one healthy volunteer. Here the strong variation of motion pattern from the apex to basis can be assessed. The results are in conformity with earlier studies investigating a few slices in 2D with 3D velocity encoding [43,44]. This 3D anatomical + 3D velocity encoded whole heart cine imaging may in future be used to classify motion abnormalities in diverse cardiac diseases such as cardiac insufficiency or asynchrony.

## Conclusions

In summary, a combination of TPM and SENSE for the acceleration of phase-encoded data acquisition is possible with only negligible motion information loss for all investigated acceleration factors. This enables a decrease of scan duration of 75% or increase of volume coverage of 4.

The SENSE sequence has the potential to enable TPM measurements in clinical routine and 3D whole heart TPM measurements in reasonable image acquisition times. The possible combination of SENSE with imaging techniques using correlations in k-space and time like k-t BLAST may offer a way to further reduce the overall scan time.

## Competing interests

VR and AL have a research grant with Philips Healthcare. PE is employed by Philips Healthcare. RM is employed by Philips Research.

## Authors' contributions

AL developed the sequence protocol, performed the data acquisition, the analysis and interpretation of data and drafted the manuscript. AB was involved in developing the sequence an in interpreting the data. PE was was involved in the analysis of data. RM and GUN were involved in the interpretation of data. VR and AB made substantial contributions to conception and design. VR, AB and WR revised the manuscript critically for important intellectual content. All authors have given final approval of the version to be published.
